# Anti-Cancer Strategies Using Anaerobic Spore-Forming Bacteria *Clostridium*: Advances and Synergistic Approaches

**DOI:** 10.3390/life15030465

**Published:** 2025-03-14

**Authors:** Saloni Singh, Geun-Hyung Kim, Kwang-Rim Baek, Seung-Oh Seo

**Affiliations:** 1 Department of Food Science and Biotechnology, Seoul National University of Science and Technology, 232 Gongneung-ro, Nowon-gu, Seoul 01811, Republic of Koreagh.kim@seoultech.ac.kr (G.-H.K.); rimmy@seoultech.ac.kr (K.-R.B.); 2 Research Institute of Food and Biotechnology, Seoul National University of Science and Technology, Seoul 01811, Republic of Korea

**Keywords:** cancer, *Clostridium*, hypoxia, bacterial-cancer therapy, synthetic biology

## Abstract

Despite ongoing advancements, cancer remains a significant global health concern, with a persistent challenge in identifying a definitive cure. While various cancer therapies have been developed and approved, offering treatments for smaller neoplasms, their efficacy diminishes in solid tumors and hypoxic environments, particularly for chemotherapy and radiation therapy. A novel approach, *Clostridium*-based therapy, has emerged as a promising candidate for current solid tumor treatments due to its unique affinity for the hypoxic tumor microenvironment. This review examines the potential of *Clostridium* in cancer treatment, encompassing direct tumor lysis, immune modulation, and synergistic effects with existing cancer therapies. Advancements in synthetic biology have further enhanced its potential through genetic modifications, such as the removal of alpha toxin gene from *Clostridium novyi*-NT, the implementation of targeted approaches, and reduction in systemic toxicity. Although preclinical and clinical studies have demonstrated that *Clostridium*-based treatments combined with other therapies hold promise for complete cancer eradication, challenges persist. Through this review, we also propose that the integration of various methods and technologies together with *Clostridium*-based therapy may lead to the complete eradication of cancer in the future.

## 1. Introduction

Cancer remains the leading cause of death worldwide, accounting for 10 million deaths annually [1]. Previous studies have shown that tumors are more than just proliferating insular masses, and the tumor environment plays a very important role. The hallmarks of cancer, as explained by D. Hanahan in 2022, have a solid foundation for understanding the biology of cancer [2]. The type of treatment depends on the stage of advancement and the type of cancer. Advancement in science and technology has provided various treatments of cancer, such as biomarker testing, which looks for genes, proteins, and other makers called tumor markers [3]; chemotherapy, which uses drugs to kill cancer cells [4]; hormone therapy, which slows the growth of breast and prostate cancer cells [5]; hyperthermia, which uses high heating to kill cancer cells [6]; immunotherapy, which helps the immune system to fight cancer cells [7]; photodynamic therapy, which uses light to kill cancer and abnormal cells [8]; radiation therapy, which uses high doses of radiation to kill cancer cells [9]; stem cell transplants consisting of procedures to restore stem cells that were destroyed through high doses of chemotherapy; surgery removing the cancer from the body [10]; and targeted therapy, which is the therapy that targets the changes in the cancer cells [11].

In the course of these cancer treatments, there are many challenges and other health complications that arise as post-effects. However, surgery is invasive and requires precision. In the brain, it can impair function and may lead to disability [12]. Radiation therapy may damage healthy cells, causing skin irritation and increasing the risk of secondary cancer. Over time, chemotherapy may lead to cancer cell resistance, and prolonged sessions can lead to heart or kidney toxicity [9]. Immunotherapy can trigger autoimmune systems that affect healthy organs [13]. Cancer cells may adapt to targeted therapy over time, leading to resistance, and hormone therapy may lead to certain health conditions due to an imbalance in hormone levels. Stem transplants may carry graft-versus-host disease (GVHD) if the donor cells attack the patient’s body [11].

Microbial therapy is a compelling alternative to overcome these side effects. Some bacteria are drawn to the hypoxic environment inside tumors and can be engineered precisely to selectively target cancer cells that are difficult to achieve with radiation or chemotherapy. Bacterial therapy provides deep penetration through the dense structure of tumor masses. Certain prominent bacteria, such as *Clostridium*, *Salmonella*, *Listeria*, and *Bifidobacterium*, are naturally capable of infiltrating and thriving within these tumor masses, making them effective for solid tumors and have entered the clinical stages [14]. However, apart from these benefits, there are safety concerns as they pose a risk of infection, particularly in immunocompromised patients. In addition, balancing the immunostimulatory effects of bacteria without causing excessive inflammation or autoimmune reactions can be challenging. To date, *Mycobacterium bovis* Bacille Calmette-Guerin (BCG) is the only bacterial mediated therapy that has been approved by the FDA for bladder cancer treatment [15]. In addition, other bacteria, such as *Pseudomonas* [16], *Streptococcus* [17,18], *Lactobacillus* [19], and *Caulobacter* [20], have been harnessed for their ability to inhibit solid tumors.

However, the capacity of *Salmonella* and *Clostridium* species such as *S. enterica* serovar Typhimurium [21] and *C. novyi* [22] to selectively colonize the tumor microenvironment (TME) makes them promising for use in cancer treatment. The initial transfer of bacteria to tumors and normal tissues is frequently comparable; however, because of the immunosuppressive and biochemically distinct TME, bacteria in tumors multiply widely, whereas those in normal tissues are rapidly eliminated [23]. By infecting immune cells such as myeloid-derived suppressor cells (MDSCs), which selectively transfer bacteria to the TME, *Listeria monocytogenes* employ a distinct method that protects tumors from immune clearance but not healthy tissues [24,25]. *Bifidobacterium longum* is an anaerobic bacterium that can colonize hypoxic environments but cannot form spores, is difficult to store and handle, and is more susceptible to unfavorable conditions [26]. It is important to note that anaerobic bacteria such as *Clostridium* do not colonize non-cancerous hypoxic or inflammatory lesions [27]; instead, they exclusively target hypoxic tumor regions. Therefore, this review explores the potential for diverse therapeutic mechanisms focusing on the ability of *Clostridium* to selectively lyse the tumor cells in the hypoxic environment. In addition, this review also provides insights into combining conventional therapies with *Clostridium* to completely eradicate tumors in future.

## 2. Biological Characteristics of *Clostridium*

### 2.1. Anaerobic Nature and Tumor Targeting Through Spore Formation

**Figure 1:**
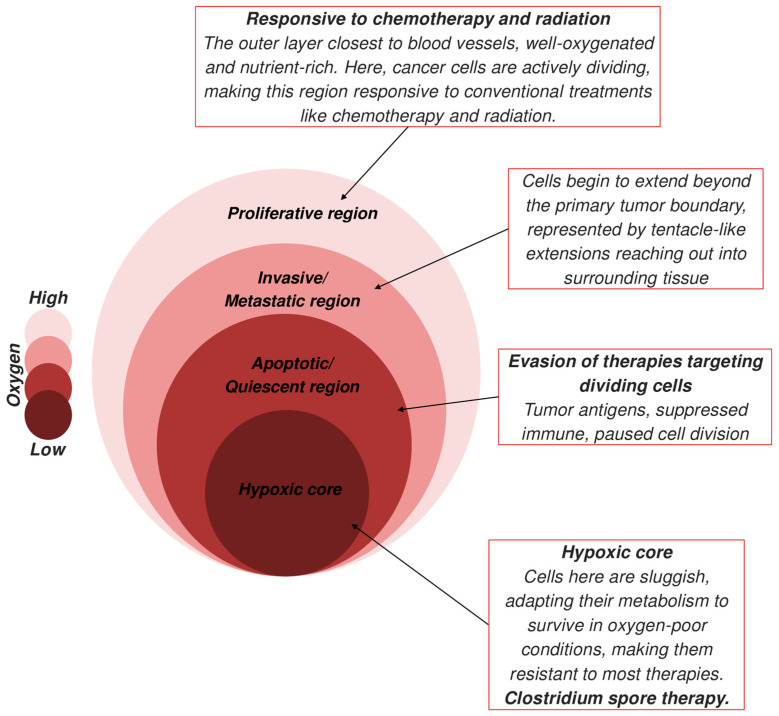
Schematic representation of tumors with different oxygenation levels and varying levels of responsiveness to therapy. The different regions in the tumor have different levels of oxygen, which makes the chemotherapy and radiation therapy effective. As the depth of the tumor increases, the oxygen level decreases as the distance from the blood vessels increases, making traditional therapies ineffective. *Clostridium* spore therapy can work on solid cores owing to its anaerobic and spore-forming capabilities.

Tumors have various zones and are structurally complex. They have proliferative regions near blood vessels containing active dividing cancer cells that respond to conventional treatments. The outer edges have invasive and metastatic regions, where the cells break and invade the surrounding tissues. The apoptotic regions develop where there is nutrient deprivation, and the surrounding quiescent regions are where cells enter dormancy due to limited resources. Traditional methods stop working and pose a threat that they may re-enter the cell cycle. At the core lies hypoxic regions with severely low oxygen resulting from inadequate blood supply. This region adapts to slow metabolism, making it resistant to conventional therapies such as radiation therapy, which relies on oxygen to damage DNA (Figure 1). *Clostridium* is an obligatory anaerobe that can germinate in areas with low oxygen levels, such as the central portions of solid tumors [28]. This property of *Clostridium* may facilitate the eradication of large, untreatable tumors that radiation, drugs, or facultative anaerobes may not reach.

### 2.2. Pathogenic vs. Therapeutic Strains

*Clostridium* species are a diverse group of anaerobic spore-forming bacteria that can vary from infamous pathogens to potentially useful medicinal substances. Strong exotoxins are produced by pathogenic strains of *Clostridium*, including *Clostridium difficile*, *Clostridium botulinum*, and *Clostridium tetani* [29,30,31]. These organisms cause tetanus, botulism, and severe gastrointestinal illnesses, respectively, but have also been tested for their anticancer abilities. Some strains, such as *Clostridium acetobutylicum* DSM792, have been modified to express therapeutic chemicals, such as tumor necrosis factor (TNF-α) and interleukin-2 (IL-2), which further enhance anti-tumor immunity and have direct lethal effects in the tumor microenvironment by inducing T cells [32]. CT26 colon cancer cells and B16–F10 melanoma cells treated with *C. difficile* toxin B (TcdB) have been found to cause an extended tumor-specific immune response helpful in the inhibition and detection of cancer [33].

In hypoxic tumor regions, *Clostridium novyi*-NT, an engineered variant of *C. novyi* that does not contain the deadly α-toxin, has shown the capacity to target and lyse cancer cells while also inducing a strong immune response by attracting immune cells and releasing inflammatory cytokines. *C. novyi*-NT has been engineered using a heavy-chain subclass of antibodies (VHH) targeting HIF1α [34]. The response is rare, but it can be combined with other chemotherapeutic agents or radiation, called Combination Bacteriolytic Therapy (COBALT), for greater efficiency, as discussed in detail below. It has also been tested as an alternative for immunotherapy, as it induces cytokines such as MIP-2, TIMP-1, G-CSF, and IL-6, leading to long-lasting anti-tumor activity [34,35]. Although the exact mechanisms by which *C. novyi*-NT eliminates tumors are unknown, the immune response to *C. difficile* toxin B may provide some insights [33]. *C. sporogenes* is also a non-pathogenic strain that has been extensively used in prodrug therapy. Notwithstanding these encouraging uses, several obstacles still exist, such as the possibility of unchecked bacterial growth, toxicities linked to the immune system, and technical difficulties of gene-editing *Clostridium* to improve tumor selectivity without reintroducing harmful characteristics. The fact that *Clostridium* is both a pathogen and a medicinal agent underscores the need for precise biotechnological control, balancing its ability to target and destroy tumors with stringent safeguards against adverse effects in clinical settings [36].

## 3. *Clostridium*-Based Anti-Cancer Mechanisms

### 3.1. Direct Tumor Lysis

Directed tumor lysis relies on the ability of non-toxic *C. novyi*-NT to selectively target hypoxic areas and cause the lysis of cancer cells. Studies in rats and canines with spontaneous tumors have shown that these bacteria localize to the tumor tissue, sparing healthy cells due to their strict anaerobic environments. To date, two routes of administration have been studied, i.e., intratumoral and intravenous administration. Upon intratumoral injection, the spores initiated a strong inflammatroy response, attracting the immune cells to the site and leading to an amplification of the immune-mediated attack on the tumor [37]. Although the exact mechanisms by which the anti-tumor function works are not fully established, it could be mostly due to the dysregulation of enzymes and the elevation of host immune responses, such as the recruitment of T cells [38]. The intravenous administration might have some advantages on solid tumors by targeting various tumors and activating natural immune responses [37]. By secreting several proteins, including phospholipase C(PLC) (NT01CX0979) and two lipases (NT01CX2047 and NT01CX0630), *C. novyi*-NT directly affected the lipid layer of the host cell wall (Table 1). These changes in the lipid bilayer structure and membrane permeability result in direct cytotoxicity, which causes tumor lysis and immune responses [39].

### 3.2. Immune Modulation

The administration of *C. novyi*-NT altered the TME. The enzymes secreted, such as phospholipase C, are also capable of eliciting immune responses [39]. In another study, dogs with naturally developing neoplasia received sopres of *C. novyi*-NT. After intratumoral injection, there is an increase in the production of LPS-induced TNF-α, LTA-induced IL-10, and NK cell function [22]. The local buildup of granular leukocytes, primarily neutrophils, has been found to restrict bacterial dissemination within the tumor, thereby limiting full oncolytic activity [40]. By selectively depleting neutrophils, either through anti-Ly6G antibody treatment or bone marrow suppression with hydroxyurea, there was a significant increase in tumor clearance; when this strategy was applied to rabbits with aggressive intracranial brain tumors, the majority achieved long-term survival without notable toxicity [40]. Therefore, *C. novyi*-NT-mediated immunotherapy is remarkably effective for cancer treatment (Table 1).

### 3.3. Combination of Clostridium with Other Therapies

*C. novyi*-NT, an obligate anaerobe, can only proliferate in oxygen-deprived environments, resulting in limited efficacy when utilized independently. The tumor microenvironment comprises diverse oxygenated regions (Figure 1) that exhibit resistance to *C. novyi*-NT but remain susceptible to alternative therapeutic modalities such as chemotherapy, radiation therapy, immunotherapy, TACE, and COBALT [41,42]. Radiotherapy, which relies on oxygen to generate DNA-damaging reactive oxygen species, can be enhanced by the capacity of *C. novyi*-NT to target and eliminate hypoxic regions, thereby improving overall tumor control. Another example is the COBALT, which incorporates modified *Clostridium* to exhibit antitumor properties, such as proteolytic enzymes, administered in conjunction with other conventional therapies synergistically. *C. novyi*-NT exhibits oncolytic properties, and its lipid-degrading enzymes are highly expressed in solid tumors. Consequently, its spores, combined with a liposomal formulation of doxorubicin called Doxil, were utilized and completely eradicated tumors [43]. Liposome-mediated prefrenctial release of drugs has also been a successful alternative therapy. Significant results have been observed when *C. novyi*-NT spores were introduced in combination with microtubule-interacting chemotherapeutic agents, including vinorelbine and docetaxel [44]. *Clostridium* has also been combined with vascular targeting agents such as CombrestainA4-phosphate. This agent targets and disrupts blood vessels, which induces further hypoxia in tumors, thereby facilitating increased colonization of *Clostridium* in smaller tumors. This approach has demonstrated enhanced efficiency of *Clostridium*-mediated delivery systems [45].

**Table 1:** Characteristics of *Clostridium* species and mechanisms of action exploited for cancer treatment.

***Clostridium*** **-Based Approach**	**Mechanism**	**Description**	**Applications**	**References**
**Spore injection**	Selective germination in hypoxic tumor regions	*Clostridium* spores germinate specifically in low-oxygen zones, proliferating and causing tumor cell lysis.	Shown to reduce tumors in models and early human trials using *Clostridium novyi*-NT spores.	[41,46]
***Clostridium*** ***novyi*** **-NT (Engineered)**	Attenuation to remove toxins, tumor-specific germination	Genetically modified to eliminate harmful alpha-toxins, allowing safer intratumoral proliferation.	Demonstrated tumor shrinkage in animal models and Phase 1 clinical trial in a patient with leiomyosarcoma.	[46]
**Enzyme Prodrug Therapy (CDEPT)**	Enzyme activation of prodrugs into cytotoxic agents within the tumor, (“bystander effect”)	Genetically engineered *Clostridium* expresses enzymes that convert non-toxic prodrugs into active chemotherapy agents only within the tumor, limiting systemic toxicity.	Studies with prodrug-converting enzymes like nitroreductase show promising local cancer cell death.	[47]
**Proteolytic activity**	Direct breakdown of tumor tissue through enzymes	Some strains like *C. sporogenes* and *C. novyi* exhibit inherent proteolytic action, which aids in breaking down the tumor extracellular matrix, increasing the efficacy of tumor lysis and immune response.	Used as a basis in research for combining proteolytic *Clostridium* strains with other therapeutic gene modifications.	[41,48]
**Immune activation**	Tumor cell lysis and immune response stimulation	As *Clostridium* proliferates in the tumor, it causes cell lysis that releases tumor antigens, which can then trigger immune cell infiltration and anti-tumor immune responses.	*Clostridium novyi*-NT therapy shows immune activation with inflammatory and tumor response in dog and rat models.	[47,49,50]
**Combination with radiation/** **chemotherapy**	Increased colonization and tumor hypoxia exploitation	Radiation can increase hypoxia and necrosis, creating an even more favorable environment for *Clostridium* colonization and synergistic effects with immune and tumor cell targeting.	Shown to enhance the therapeutic effects in preclinical studies when used in conjunction with chemo/radiotherapy.	[27]
***Clostridium*** **Species**	**Toxicology**	**Mode of Action**	**Genetic** **Modifications**	**References**
***C. novyi*** **-NT**	Inflammation Bacterial spread Immune reactions	Targets hypoxic tumors Causes tumor cell lysis Activates immune response	α-toxin gene removed for safety	[22]
***C. perfringens***	Intestinal damage Inflammation Bacterial spread	Produces enterotoxin (CPE)	Usage of c-terminal fragment of CPE (c-CPE)	[51,52]
***C. sporogenes***	Immune clearance Bacterial spread	Anaerobic tumor colonization Activates prodrugs	Engineered for prodrug activation (e.g., overexpressing NTR enzyme)	[53]
***C. difficile***	High toxicity (Toxin A and B) Gut inflammation Bacterial spread	Potential tumor targeting but mainly produces toxins	Not suitable for therapy	[54]

## 4. Synthetic Biology for *Clostridium*-Based Anti-Cancer Therapies

### 4.1. Genetic Modification

Gene therapy involves the incorporation of correct genetic material into target cells to achieve therapeutic effects. Genetic modification has enabled the development of multiple therapeutic approaches. Various efforts have been made to exploit *Clostridium* for its hypoxic ability. The most prominent example of gene modification is the α-toxin in *C. novyi*. This toxin has been removed to create a non-toxic strain, denoted as NT in *C. novyi*-NT. This modification facilitates the successful colonization of tumor cells in oxygen-deprived regions while preserving nearby healthy cells. Gene modification has also enabled immunotherapy using *Clostridium*. Recently, *Clostridium*-directed antibody therapy (CDAT) has gained prominence to produce high-specificity antibodies against tumor antigens through gene modification. In a recent study, *C. acetobutylicum* DSM792 was engineered to express and secrete TNF-α. However, due to the low colonization of this bacterium, no therapeutic results were obtained. Nonetheless, IL-2 expression successfully reduced tumors while avoiding systemic toxicity [32].

In theory, numerous approaches exist for engineering bacteria to achieve promising outcomes. However, multiple practical challenges also exist. *Clostridium* species such as *C. acetobutylicum*, while readily transformable due to their less complex genetic structure, exhibit low colonization rates, potentially due to limited adaptability to host environments. Conversely, species such as *C. novyi*-NT, *C. oncolyticum*, and *C. sporogenes* present challenges in transformation. The evolutionary adaptations, sporulation characteristics, and metabolic differences play a significant role in these variations. Nevertheless, the utilization of *E. coli* as a vector for successful transformation has significantly advanced cancer therapy possibilities, owing to developments in genetic engineering techniques [53].

### 4.2. Expression of Prodrugs

Suicide gene therapy, also called gene-directed enzyme prodrug therapy, utilizes enzymes to modify a prodrug into its cancer-killing form. In the case of CDEPT, *Clostridium* serves as a vector producing prodrug-activating enzymes through genetic modification. When *Clostridium* spores are injected, they selectively germinate in the tumor hypoxic regions and produce enzymes that convert the prodrug into its active form, ensuring that the toxin is specifically released in the tumor. One of the first enzyme/prodrug systems to be engineered was the 5FC (5-fluorocytosine) and CD (cytosine deaminase) system, where CD converts 5FC to 5FU (5-fluorouracil), which is a cytotoxic compound. Other examples include Nitroreductase (NfsB, NmeNTR, sNTR, and HinNTR) and Carboxypeptidase G2 (CPG2), which have different mechanisms of action (Table 2). Although these enzymes have been cloned in various strains of *Clostridium*, the most prominent is *C. sporogenes* as promising results were obtained with NTR/PR-104 when the spores were injected in tumor-bearing mice [55]. Although prodrugs hold a promising approach, there are some negative effects to be considered as well. Some prodrugs may have a damaging effect on bacterial DNA, proteins or metabolism and may kill the bacteria before they finish the activation of prodrug. 5FU (5-fluorouracil) is a DNA synthesis inhibitor that may also affect the bacterial replication [56]. Thus, to overcome these challenges, optimization is required. One of the approaches could be encapsulation with biomaterials such as nanoparticles that can allow the bacteria to survive longer.

### 4.3. Delivery of Therapeutic Proteins

A significant challenge in genetic engineering has been the development of an effective delivery mechanism for cancer treatments utilizing *Clostridium*, ensuring appropriate secretion of therapeutic agents such as TNF-α or enzymes like CD (Cytosine deaminase) to function directly within the tumor microenvironment. *Clostridium* lacks the ability to export large proteins, prompting researchers to develop various strategies to address this limitation. One approach involves the attachment of small amino acid sequences known as signal peptides to the therapeutic proteins, as demonstrated in *C. acetobutylicum*. This species has been genetically modified to produce cytosine deaminase using the clostripain signal sequence, originally derived from *C. histolyticum*, to enhance enzyme activity [61]. An alternative method involves the fusion of promoters for controlled expression. The recA promoter, which is radio-inducible, has been employed to regulate therapeutic protein expression in response to radiation therapy. When radiation therapy induces DNA damage, this promoter increases gene expression specifically at the irradiated tumor site. The addition of “cheo boxes” (specific DNA binding sites) further enhances its sensitivity, augmenting production following radiation while minimizing baseline activity in the absence of radiation [62]. This strategy has demonstrated advantages for controlled release of anti-cancer proteins during radiotherapy sessions, improving tumor targeting and reducing off-target effects [45].

Two critical immune checkpoints utilized for the delivery of therapeutic proteins are the PD-1/PD-L1 pathway and the CTLA-4 pathway. Cancer cells produce PD-L1, a protein that interacts with PD-1 on T lymphocytes, thereby suppressing their capacity to eliminate tumor cells. To counteract this mechanism, therapeutic agents that inhibit either PD-1 (such as nivolumab and pembrolizumab) or PD-L1 (like atezolizumab) are employed. These inhibitors prevent the PD-L1/PD-1 interaction, thus enhancing the T-cell-mediated immune response against cancer. Another immune checkpoint that suppresses immune responses is CTLA-4, which interacts with B7 molecules on antigen-presenting cells to inhibit T-cell activation. To enable T cells to target cancer cells, CTLA-4 inhibitors such as ipilimumab are utilized [63]. This approach can potentially enhance the efficacy of immune modulation if *C. novyi*-NT can be genetically engineered to achieve targeted release at the tumor site.

## 5. Preclinical and Clinical Studies

### 5.1. Preclinical Models

Preclinical trials have been conducted to evaluate *C. novyi*-NT spores on various rodent models. In previous research, intracranial syngeneic F98 and human xenograft 060919 rat GBM models were inoculated with *C. novyi*-NT spores, resulting in specific tumor destruction while preserving the normal brain parenchyma [64]. Given the similarities between human and canine tumors, dogs with naturally occurring tumors have been subjected to spore therapy, leading to complete or partial tumor destruction in 37.5% of cases [64]. This study also reported some adverse effects, such as inflammation at the injection site, which were manageable with standard care. Other preclinical studies have also focused on combining immune system activation with *C. novyi*-NT [65].

### 5.2. Clinical Trials

The initial human trial (NCT01924689) was conducted in 2021 to ascertain the dose-limiting toxicities (DLTs) and maximum tolerated dose for intratumoral injection of *C. novyi*-NT regression [46]. A dose-dependent trial (1 × 10^6^ spores) was implemented across six cohorts, and it was observed that spores could germinate within the tumor tissues, inducing lysis and eliciting an immune response. Among the 24 patients, 42% experienced tumor lysis, and 86% achieved stability. The maximum tolerated dose (MTD) was determined to be 1 million spores, with some adverse effects such as sepsis and gas gangrene.

Following the successful examinations in trial I, a Phase Ib/II study is currently in progress, investigating the combination of Pembrolizumab, an immune checkpoint inhibitor (NCT03435952) [66]. The objective is to enhance the anti-tumor effect. Approximately 25% of patients have demonstrated partial tumor responses, particularly in cases of tongue squamous cell carcinoma and nasopharyngeal cancer. The most frequently observed mild adverse effects were injection site reactions, fever, and anemia, with no severe (grade 3 or 4) side effects reported. This ongoing study aims to identify the optimal dose for future trials and shows promising clinical activity with pembrolizumab [66].

## 6. Challenges and Limitations

### 6.1. Safety Concerns

**Figure 2:**
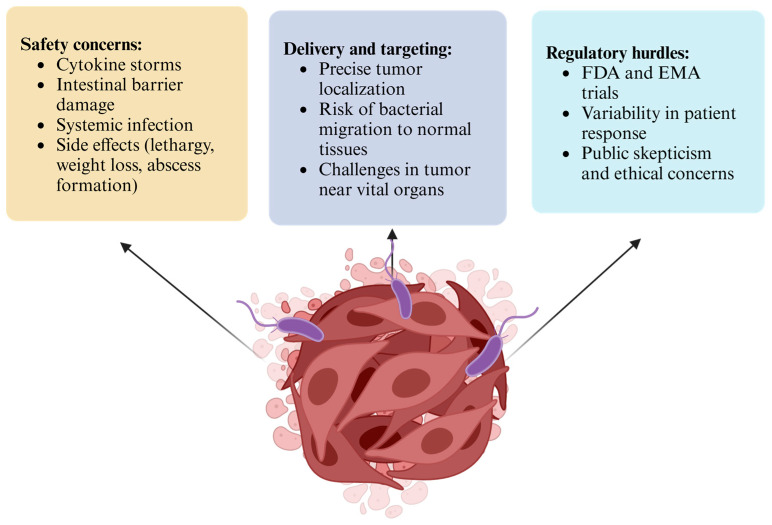
Challenges and limitations of using *Clostridium* in cancer therapy (created in https://BioRender.com).

The utilization of bacteria as a therapeutic agent presents inherent risks. Although *C. novyi*-NT has been genetically modified to lack alpha toxin, it may still elicit immune-stimulating effects. As previously noted, clinical studies have reported mild side effects that could potentially lead to adverse reactions [46]. The introduction of bacterial spores may induce a robust immune response, such as cytokine storms, wherein the immune system exhibits an exaggerated reaction. The spores may also interact with the uninfected cells, which may damage the intestinal barrier and initiate inflammation. Usually, this bacterial strain is itself not harmful but its spores may trigger some immune response (Figure 2).

There is also a significant risk that bacteria may proliferate beyond their intended therapeutic effect, potentially leading to uncontrolled bacterial growth. These microorganisms may disseminate to other body parts and potentially cause systemic infection [67]. There have been observed various toxicities related such as lethargy, weight loss, and abscessation in animal studies. In some trials, dose-limiting toxicities such as abscess formation have occurred, particularly when high doses are administered [68]; however, other study approaches have balanced the toxicity by introducing antibiotics together [64,68]. This therapeutic approach necessitates appropriate management and the establishment of a standardized dosage regimen to be considered for commercial availability.

### 6.2. Delivery and Targeting

*C. novyi*-NT proliferates in the hypoxic environment within solid tumors. However, precise delivery systems that do not affect normal tissues are essential, particularly when tumors are located in proximity to vital organs or surrounded by normal tissues. Intratumoral injections also present a potential risk due to the possibility of bacterial migration to other body parts (Figure 2). At present, strategies are under investigation to restrict bacterial presence to the tumor (intratumoral injection) and utilize physical barriers or engineered control systems to contain bacterial dissemination within the targeted area [69].

### 6.3. Regulatory Hurdles

Regulatory institutions such as the FDA in the United States and the EMA in Europe require extensive trials to ensure safety. Unlike conventional drugs, these are live bacteria that are more challenging to control, monitor, and standardize. Patient variability also contributes to the difficulty in standardizing treatment conditions. Furthermore, manufacturing and quality production issues persist (Figure 2). The production of *C. novyi*-NT at high quality and purity presents a challenge due to its complexity. It is crucial to ensure an effective and standard dose that is free of contaminants, and it is important to note that they maintain their non-toxic state. Additionally, ethical concerns and public acceptance remain subjects of skepticism [70].

## 7. Future Perspectives

### 7.1. Next-Generation Engineering

Advancements in synthetic biology have enabled the development of more precise mechanisms for engineering *Clostridium* for specific targeting. Through these precise mechanisms targeting hypoxic tumors, enhanced immune response and other combination therapies such as prodrugs and other therapeutic deliveries have become feasible. The development of the ClosTron technique, which adapted the TargeTron platform, has facilitated site-specific gene insertion into *Clostridium*. This method is based on the retrohoming of group II introns, allowing a targeted insertion of up to 0.4 kb of DNA, as well as incorporating a selective marker for precise modification [71]. This genetic tool has also been utilized for the secretion of human atrial natriuretic peptide (ANP), a peptide that can suppress inflammatory response. Upon injection into tumors in mice, *C. novyi*-NT-ANP demonstrated the ability to reduce inflammation-induced mortality and improve tumor clearance [72]. However, this genetic tool relies on selective markers and may have limitations where antibiotic resistance should be minimized. An increase in DNA payload capacity is also required to expand its applications. Additionally, as previously discussed, the combination with conventional therapies such as chemotherapy and radiation therapy could also enhance efficacy. By focusing on these aspects, next-generation engineering has the potential to significantly advance the effectiveness of *Clostridium* in oncology.

### 7.2. Personalized Medicine

The integration of personalized medicine for cancer therapy utilizing *Clostridium* has the potential to revolutionize therapeutic approaches. Individual patients exhibit variations in cancer stage, immune response, and tumor type. Personalized medicine can enhance efficacy by selecting therapies based on the genetic, molecular, and cellular characteristics of the tumor while minimizing adverse effects. Genetic variations influence an individual’s drug metabolism, thus an optimized dosage of *Clostridium* could facilitate adaptive and responsive treatment. With the advent of live biotherapeutics (LBSs), *Clostridium* can be employed in a manner specifically tailored to each cancer type and immune landscape [73]. Future studies can be performed by integrating real-time biomarker monitoring and tumor sequencing to guide the treatment decisions. The advances in biosensors may allow various adjustments to bacterial based therapy based on patient-specific responses.

### 7.3. Integration with Emerging Technologies

To significantly enhance the efficacy of cancer treatment, several advanced technologies such as CRISPR, nanotechnology, and AI-driven drug delivery systems may prove effective in eradicating cancer. Nanotechnology can facilitate targeted localization of *Clostridium* spores at the tumor site, thereby minimizing the risk of unintended bacterial dissemination. Liposomes and albumin nanoparticles are the US FDA-approved nanocarriers that have demonstrated efficacy in cancer therapy. In the case of *Clostridium*, nanoparticles can be utilized to encapsulate *Clostridium* spores, enabling controlled release and the targeting of the tumor microenvironment [74]. CRISPR enables precise modifications of the *Clostridium* genome, which is advantageous for editing toxin genes, immunotherapy, and prodrug delivery. Recently, CRISPR/Cas9-modified *C. novyi*-NT has been engineered to insert the RGD gene, a tumor targeting peptide, expressed within the promoter region of spore coat protein. This resulted in increased tumor localization upon intravenous administration and immune stimulation [75]. Furthermore, AI-driven drug discovery can expedite the identification of optimal genetic configurations and treatment combinations. For instance, AI can analyze characteristics from previous medical records and apply them to clinical practice. AI can develop pattern analysis and classical algorithms to facilitate more informed decisions on diagnosis and treatment in cancer therapy [74]. AI can also be integrated with nanotechnology to increase efficiency. This approach may prove particularly beneficial for personalized medicine. Another approach could involve the utilization of IoT (Internet of Things), which could be beneficial for remote monitoring and real-time data collection through wearable devices. Patient health metrics such as heart rate, temperature, and activity levels can be recorded based on the response to the treatment [74]. These approaches offer a pathway towards highly sophisticated, programmable cancer therapies.

## 8. Conclusions

*Clostridium*-based therapies present a promising approach for the treatment of cancer and, in conjunction with other conventional treatments such as radiation, chemotherapy, and immunotherapy, demonstrate a potential pathway for complete eradication. Advancements in synthetic biology have facilitated the targeted delivery of *Clostridium* spores to the tumor microenvironment, which is characteristically hypoxic. By enabling precise gene editing and engineering, *Clostridium* can deliver drugs, convert prodrugs, enhance immune responses, and improve efficacy while mitigating side effects. The advancement of science, introducing CRISPR and the era of artificial intelligence, represents a significant development in the field of cancer research. Their integration for optimizing treatments has demonstrated considerable potential for cancer treatment. While this approach shows promise, challenges pertaining to ensuring safety, controlling bacterial growth, and overcoming obstacles persist. Continued research and clinical trials are necessary to enhance and refine these therapies, with the aim of developing more targeted, adaptable, and safer cancer treatments.

## Figures and Tables

**Table 2:** Prodrugs used in *Clostridium*-mediated cancer therapies, their corresponding prodrug-converting enzymes (PCEs), and their resulting active metabolites.

Prodrug	Prodrug-Converting Enzyme (PCE)	Bacterial Strain	Active Metabolite	Mechanism	References
**5-Fluorocytosine (5-FC)**	Cytosine deaminase (CD)	*C. beijerinckii*, *C. acetobutylicum*, *C. sporogenes*	5-Fluorouracil (5-FU)	Inhibits DNA/RNA synthesis and sensitizes tumors to radiotherapy.	[31,57,58]
**CB1954 (Nitrobenzamide)**	Nitroreductase (NfsB, NmeNTR)	*C. beijerinckii*, *C. sporogenes*	4-Hydroxylamine (4HX)	Alkylates DNA in cancer cells, effective particularly in hypoxic conditions.	[53,59,60]
**PR-104**	Nitroreductase (sNTR, HinNTR)	*C. sporogenes*	PR-104H	Crosslinks DNA in hypoxic cells, causing cytotoxicity specific to cancer cells.	[47,55,59]
**Glutamated Benzoyl Nitrogen Mustard**	Carboxypeptidase G2 (CPG2)	*C. sporogenes*	Cytotoxic nitrogen mustard derivatives	Causes DNA damage, leading to cancer cell death.	[53]

## Data Availability

No new data were created or analyzed in this study.
